# Fungi of French Guiana gathered in a taxonomic, environmental and molecular dataset

**DOI:** 10.1038/s41597-019-0218-z

**Published:** 2019-10-16

**Authors:** Gaëlle Jaouen, Audrey Sagne, Bart Buyck, Cony Decock, Eliane Louisanna, Sophie Manzi, Christopher Baraloto, Mélanie Roy, Heidy Schimann

**Affiliations:** 1 AgroParisTech - UMR EcoFoG (AgroParisTech, CIRAD, CNRS, INRA, Université des Antilles, Université de Guyane), BP 316 - F-97379 Kourou cedex, France; 2 INRA - UMR EcoFoG (AgroParisTech, CIRAD, CNRS, INRA, Université des Antilles, Université de Guyane), BP 316 - F-97379 Kourou cedex, France; 3 Institut de Systématique, Évolution, Biodiversité (ISYEB – UMR7205), Muséum National d’Histoire Naturelle, Sorbonne Université, CNRS, F-75005 Paris, France; 4 Mycothèque de l’Université catholique de Louvain (MUCL2), Earth and Life Institute—Microbiology (ELIM), B-1348 Louvain-la-Neuve, Belgium; 5 Laboratoire Évolution et Diversité Biologique, Université Toulouse 3 Paul Sabatier - CNRS, UMR 5174 UPS CNRS ENFA IRD, F-31062 Toulouse cedex 9, France; 6 Department of Biological Science, Florida International University, Miami, FL 33199 USA

**Keywords:** Biodiversity, Tropical ecology, Fungal ecology

## Abstract

In Amazonia, the knowledge about Fungi remains patchy and biased towards accessible sites. This is particularly the case in French Guiana where the existing collections have been confined to few coastal localities. Here, we aimed at filling the gaps of knowledge in undersampled areas of this region, particularly focusing on the Basidiomycota. From 2011, we comprehensively collected fruiting-bodies with a stratified and reproducible sampling scheme in 126 plots. Sites of sampling reflected the main forest habitats of French Guiana in terms of soil fertility and topography. The dataset of 5219 specimens gathers 245 genera belonging to 75 families, 642 specimens are barcoded. The dataset is not a checklist as only 27% of the specimens are identified at the species level but 96% are identified at the genus level. We found an extraordinary diversity distributed across forest habitats. The dataset is an unprecedented and original collection of Basidiomycota for the region, making specimens available for taxonomists and ecologists. The database is publicly available in the GBIF repository (10.15468/ymvlrp).

## Abstract (summary)

**Table:** 

Measurement(s)	taxonomic diversity assessment by targeted gene survey • Fungi
Technology Type(s)	Barcode-Seq • Taxonomy
Factor Type(s)	geographic location
Sample Characteristic - Organism	Fungi
Sample Characteristic - Environment	tropical lowland evergreen broadleaf rain forest
Sample Characteristic - Location	French Guiana Region

Machine-accessible metadata file describing the reported data: 10.6084/m9.figshare.9914138

## Background & Summary

Neotropical rainforests are poorly described when it comes to the Fungi. The distribution of the known species remains patchy, biased towards accessible sites^1,2^ and their ecology is still largely fragmentary^3^. In Amazonia, the interest in Mycology goes back to the 19th century, with Montagne and Leprieur who drew a first checklist of Fungi around Cayenne, French Guiana^4^ (and Berkeley^5,6^ for the Brazilian part). Since then, Amazon rainforests have been explored in their Brazilian part with important contributions by Hennings^7^ and Rick^8^ at the very beginning of the 20th century, and more recently by Singer^9,10^, Trieveiler-Pereira^11^, Sulzbacher^12^ or Ryvarden^13^. Great contributions have also been made by Henkel and collaborators^14,15^ in the Pakaraimas mountains in Guyana (www.tropicalfungi.org) or in Colombia, especially in the terra-firme and white-sand forests^16–18^. The last checklist for French Guiana (1996) listed 625 taxa^19^ gathered in a very limited number of coastal localities. Evidently, there is an urgent need to systematically collect and document fungi from undersampled areas to fill the knowledge gaps in a region where fungal diversity may be much higher than presently known^20–22^.

**Fig. 1:**
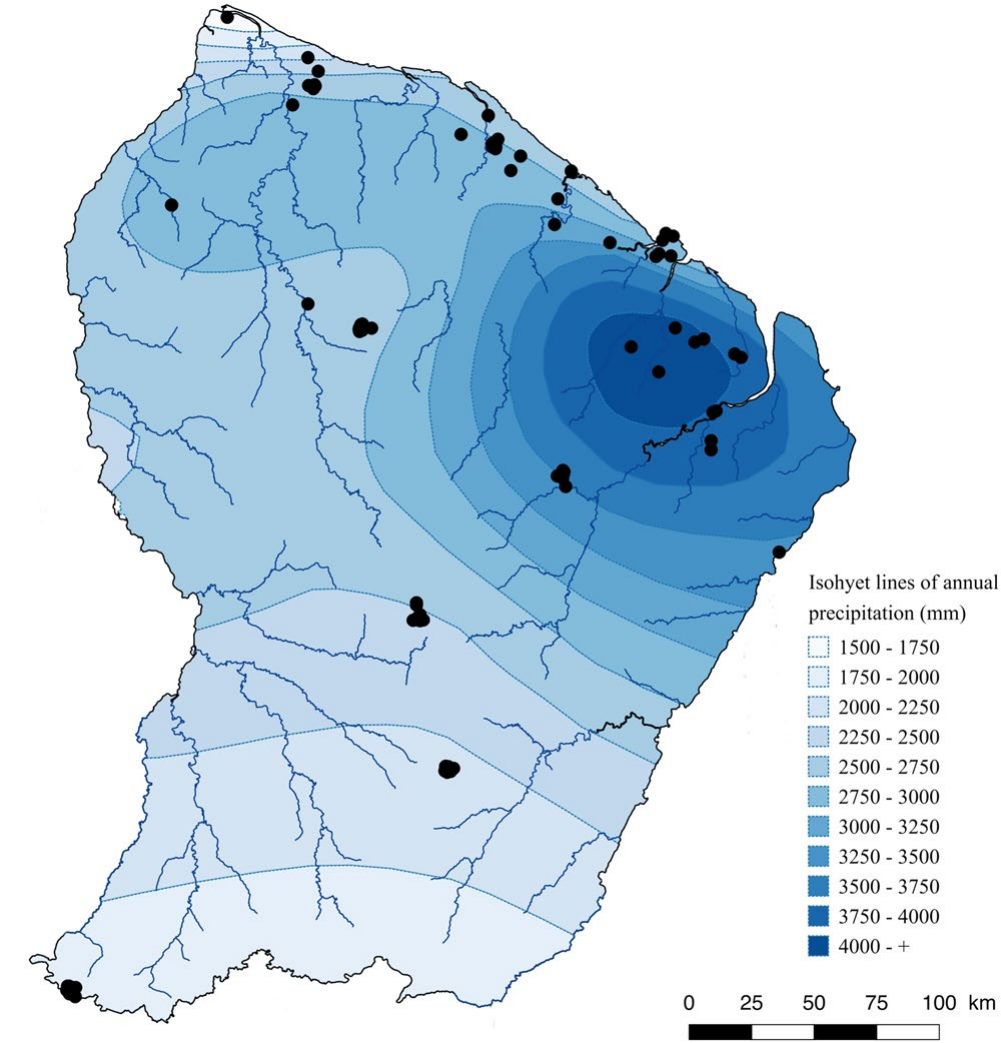
Sampling sites across French Guiana. This map of French Guiana shows the distribution of the sampling sites (black dots) represented in the dataset described in this article. Isohyet lines of precipitation are shown for information.

From 2011 onwards, we collected all fruiting-bodies following the same protocol in 126 plots representative of the main forest habitats of French Guiana (Fig. 1). We also gathered information on habitats, environment and first taxonomic indications. The resulting dataset provides an unprecedented collection of Basidiomycota for the region, making specimens available for taxonomists, with a molecular barcode for some of them, together with information on ecology and distribution.

**Fig. 2:**
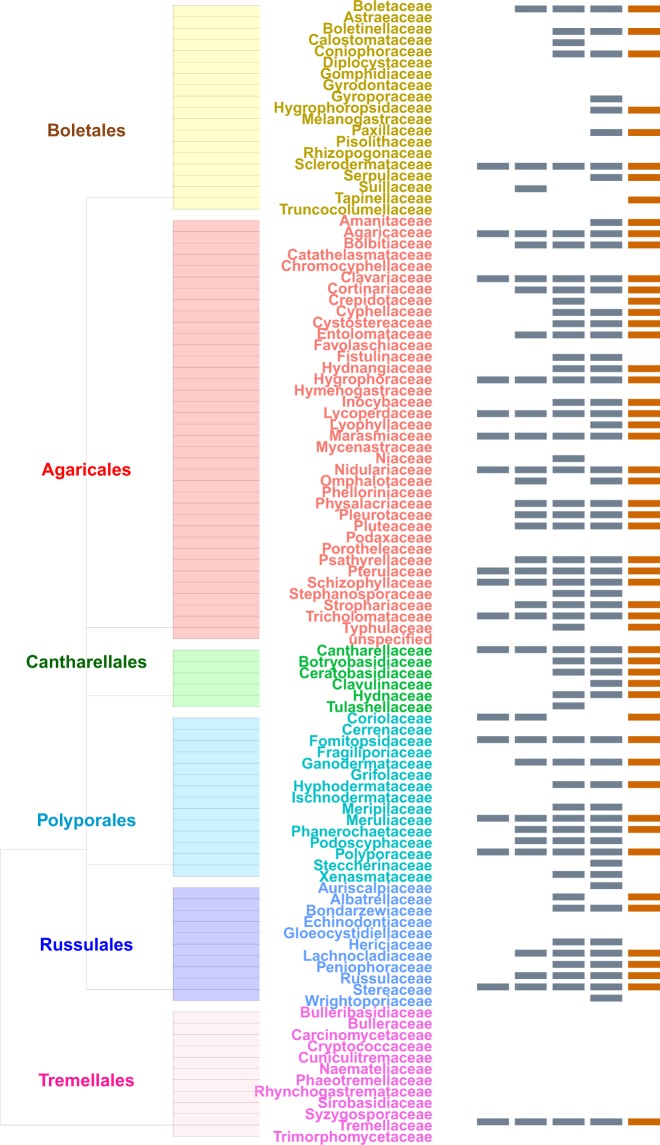
Successive contributions of sampling. Cladogram showing the contribution of: (from left to right) Montagne^4^, Courtecuisse^19^, Vasco-Palacios^16^, Species Link for Amazonia (splink.cria.org.br, 2019) and this dataset (2019) in gathering specimens. For convenience, only orders that have been more intensively sampled are displayed. We followed the classification proposed by Tedersoo *et al*.^37^.

We found an extraordinary diversity across forest habitats. The dataset is not a checklist as only 27% of the specimens are determined at the species level and 96% at the genus level. However, the voucher specimens are deposited to herbaria, mainly the Fungarium of the Paris Natural History Museum, for further identification if needed. This tedious, on-going sampling increases the number of families and genera known for the territory as compared with previous collections^4,19^ (Table 1 and Fig. 2). The number of species reported from French Guiana increased from 625^23^ to 1168. The most abundant families found in French Guiana are also among the most abundant ones found in Amazonian Brazilian forests (http://splink.cria.org.br/, keywords search = Basidiomycota AND Acre, Amapa, Amazonas, Para, Roraima, 16793 records). Around one quarter of the genera we observed were also recorded in the Amazonian part of Colombia by Vasco-Palacios and Franco Molano^16^ (Fig. 2). They recorded 119 species belonging mainly to *Tricholomataceae* (Agaricales) and *Coriolaceae* (Polyporales). Thirteen genera of ectomycorrhizal fungi (all of which were also recorded from monodominant leguminous forests in Guyana^14^, e.g. *Amanita*, *Cantharellus* or *Russula*) were, although scarce, present in several sites, thereby confirming that ectomycorrhizal fungi can persist in hyperdiverse Neotropical forests^1,22^.

**Table 1:** Quantities of orders, families and genera inventoried according to sources and geographical areas.

	Sources	Orders	Families	Genera
French Guiana	Montagne (1855)	12	26	39
	Courtecuisse (1996)	15	48	126
	This dataset (2019)	19	75	245
Amazonia	Species Link for Amazonia and This dataset (2019)	**29**	**100**	245
	Vasco-Palacios (2017)	23	82	**337**

Sources are referenced in the text. The biggest values are highlighted.

## Methods

### Geographic coverage

French Guiana (83,534 km^2^) is a French overseas region situated in South America at the eastern limit of the Guiana Shield, a mountainous tableland extending, from West to East, across Guyana, Suriname, French Guiana, as well as parts of Colombia, Venezuela and Brazil. Soils are ancient, heavily eroded and chemically poor. The country’s relief is fairly flat, rarely exceeding 200 m with three mountain chains reaching up to 830 m at Mount Itoupé^24^. The climate is characterized by a clear seasonal pattern: a wet season from December to July, which is normally interrupted in February or March by a short dry period, and a long dry season from August to November with monthly precipitation of less than 100 mm. Average annual precipitation is 2200 mm. Mean temperature is 25 °C with low seasonal changes^25^.

### Study extent

From 2011, the authors collected sporocarps in French Guiana and inventoried a total of 126 1-ha plots (Fig. 1), 6 of which had previously been visited by R. Courtecuisse^19^. The Nouragues Ecological Research Station (4°05′N–52°41′W, www.nouragues.cnrs.fr) and the Experimental Station of Paracou (5°18′N–52°53′W, http://paracou.cirad.fr/) are research stations where permanent forest plots are monitored for their vegetation, climate and environmental data. Limonade, Itoupe, Mitaraka are part of the National Amazonian Park of French Guiana (PAG, www.pag.fr). Kaw, Laussat and Trinite are part of the Network of Natural Reserve of French Guiana on the coast (www.guyane-parcregional.fr). CSG is located within the area under the control of Guiana Space Center (www.cnes-csg.fr). Other sampling sites were chosen because they represented several typical French Guiana’s habitat types, as defined by Guitet *et al*.^24^: margin of inselbergs, white-sands forests, terra-firme forests, seasonally flooded forests. The plots are at altitudes ranging from 35 to 800 m.

The 126 plots were assigned to one of three topographies according to the classification of Ferry *et al*.^26^: plateau if the plot is situated on upper part of hill with vertical water drainage; slope if the plot is situated along a slope and exhibited a superficial lateral drainage, and seasonally flooded if the plot is situated in a bottomland regularly inundated during rainy season with a water table always observed above 60 cm depth and present at the surface soil for at least two consecutive months^26^. Two main types of soils were selected. First, clay-rich soils or terra-firme are sand-silt-clay mixture of soils very commonly found in French Guiana^27,28^. Second, white-sand soils are soils derived from podzols as well as quartzites and weathered granite on the margin of the inselbergs according to the definition given by Baraloto *et al*.^28^.

### Sampling description

We developed an easily and reproducible field experimental procedure to collect and identify fruiting bodies. Each sampling site coordinates were recorded and associated with the World Geodetic System 1984 (WGS 1984) and UTM 21-22N for map projection. We took advantage of pre-existing 1-ha botanical plots to carry out inventories and proceeded as follows: we randomly positioned three sub-plots of 20 × 20 m in each main 1 ha-plot where two collectors exhaustively sampled all visible sporocarps, for a period of 1.5 h maximum per sub-plot. Hypogeous fungi were not targeted during these inventories. All visible sporocarps were photographed, numbered and dried using a field drier the same day and a ~0.5 cm^2^ tissue sample of each sporocarp was stored for DNA in CTAB (2% Cetyltrimethylammonium bromide).

### Taxonomic identification

The dataset of 5219 specimens gathers 245 genera belonging to 75 families. Species names of the closest morphospecies were assigned by M. Roy in the field based on existing literature^9,11,14,16,29^. Then, more precise taxonomic identification of all fungi collected was done in collaboration with R. Courtecuisse, C. Decock, T. Henkel, P.-A. Moreau, M. Roy, S. Welti, G. Grühn, J. Fournier, C. Lechat in the field or later by examination of vouchered specimens by A. Verbeken, F. Wartchow and B. Buyck, and using existing literature^29^. Homogeneity and consistency of all taxonomic names were controlled afterward. All dry voucher specimens were deposited at one of the following herbaria: LIP herbarium (Lille, Université de Lille, Département de Botanique); PC herbarium (Mycological herbarium of the Paris Natural History Museum, Paris); MUCL, Catholic University of Louvain; HSC, Humboldt State University.

### Barcoding

Among the collection, 771 specimens were barcoded as followed. DNA was extracted using the CTAB method^30^, the internal transcribed spacer (ITS1f-ITS1r primers from Taberlet *et al*.^31^) was amplified by PCR and sequenced using Illumina Miseq technology (2 × 250 bp) by Fasteris (Plan-les-Ouates, Switzerland) or at the Genotoul platform (www.genotoul.fr). We used tagged primers to distinguish sequences from each specimen. Raw data of the Illumina sequencing were analyzed with the OBITools package^32^ as well as scripts in R^33^. Briefly, we first conducted paired-end read assembly, read assignment to samples and read dereplication. Low-quality sequences, i.e. those shorter than expected (under 80 bp), containing ambiguous nucleotides, corresponding to singletons and displaying low score paired-end alignments were excluded from the analysis. Scores of pairwise alignments were calculated with Sumatra package (www.metabarcoding.org) which uses the same clustering algorithm as UCLUST and CD-HIT. This algorithm is mainly useful to detect the ‘erroneous’ sequences created during amplification and sequencing protocols, deriving from ‘true’ sequences. For each sample, sequences having pairwise alignments with a score below 97% of similarity were removed and considered as erroneous. Last, for each specimen, the most abundant sequence was kept as representative of the specimen. The last UNITE dataset (https://unite.ut.ee/) was used as reference for the taxonomic assignment of specimen target sequence. The molecular assignation was then compared to the morphological one to confirm the identification of the sequence. In case of discrepancy between the morphological and the molecular identification, the sequence was discarded. At the end, 642 sequences (140 to 256 bp length) were submitted to GenBank. The barcoding of remaining specimens is ongoing with the aim to sequence at least one specimen of each species or genus.

## Data Records

The dataset contains a record for each sample. Each record contains a unique code identifying the specimen in the collection; a code attributed by the herbarium where it is deposited; a name corresponding to the most precise identification by one of the mycologists involved in this work; the name of the specialist who identified it; the complete description of the sampling plot (city, site, plot, geographical coordinates, altitude, habitat, topography, soil type, substratum and host, name of the collector; collection date), the barcode name (ITS1), the obtained sequence for this barcode and the GenBank accession number of the barcode.

The dataset is managed locally in a shared database and is accessible publicly in the GBIF repository (www.gbif.org) under the 10.15468/ymvlrp ^34^. Updates of the online dataset are planned when major changes will occur. All unique ITS1 barcodes (642) were submitted to GenBank under accession numbers MF038887^35^ to MK547056^36^.

## Technical Validation

Homogeneity and consistency of all taxonomic names were controlled afterward thanks to MycoBank (http://www.mycobank.org) and Index Fungorum (http://www.indexfungorum.org).

**Fig. 3:**
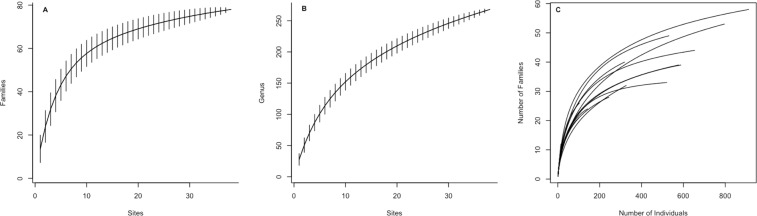
Sampling accumulation curves. (**A**) At the Family level and (**B**) at the Genus level. (**C**) Represents rarefaction curves for each sampling site. In (**A**) the flattened curve shows that we sampled the majority of Basidiomycota families present in French Guiana. On the contrary, curve in (**B**) does not really flatten, indicating that, despite our sampling effort, we probably missed some Basidiomycota genera, and underlying the crucial necessity to continue this collection. (**C**) Shows an unbalanced sampling effort by sites, probably indicating differences in species richness across contrasting sites but also differences in sampling effort. Accumulation curves were performed using Coleman method.

The dataset described in this work was gathered thanks to a field experimental procedure to collect Basidiomycota fruiting bodies exhaustively across several typical French Guiana’s habitat types. We analyse the efficiency of this sampling method by building accumulation curves (Fig. 3). These curves show that we sampled the majority of Basidiomycota families present in French Guiana. But these curves also indicate that, despite our sampling effort, we probably missed some Basidiomycota genera. This underlies the crucial necessity to continue this collection.
